# Alternating hemiplegia of childhood: evolution over time and mouse model corroboration

**DOI:** 10.1093/braincomms/fcab128

**Published:** 2021-06-04

**Authors:** Julie Uchitel, Keri Wallace, Linh Tran, Tavis Abrahamsen, Arsen Hunanyan, Lyndsey Prange, Joan Jasien, Laura Caligiuri, Milton Pratt, Blaire Rikard, Carmen Fons, Elisa De Grandis, Aikaterini Vezyroglou, Erin L Heinzen, David B Goldstein, Rosaria Vavassori, Maria T Papadopoulou, Isabella Cocco, Rebecca Moré, The Duke AHC Research Group, The French AHC Consortium, Alexis Arzimanoglou, Eleni Panagiotakaki, Mohamad A Mikati

**Affiliations:** 1 Division of Pediatric Neurology and Developmental Medicine, Department of Pediatrics, Duke University, Durham, NC 27710, USA; 2 Department of Statistical Science, Duke University, Durham, NC 27708, USA; 3 Department of Child Neurology, Sant Joan de Déu Children’s Hospital, Member of the ERN EpiCARE, Barcelona 08950, Spain; 4 Child Neuropsychiatry Unit, IRCCS Istituto Giannina Gaslini, Genoa 16147, Italy; 5 Department of Neurosciences, Rehabilitation, Ophthalmology, Genetics, Maternal and Child Health (DINOGMI), University of Genoa, Genoa 16147, Italy; 6 Department of Developmental Neurosciences, UCL NIHR BRC Great Ormond Street Institute of Child Health, London WC1N 3JH, UK; 7 Eshelman School of Pharmacy, University of North Carolina at Chapel Hill, Chapel Hill, NC 27599, USA; 8 Institute of Genomic Medicine, Columbia University, New York, NY 10032, USA; 9 Euro Mediterranean Institute of Science and Technology I.E.ME.ST, Palermo 90139, Italy; 10 Department of Pediatric Clinical Epileptology, Sleep Disorders and Functional Neurology, Member of the ERN EpiCARE, University Hospitals of Lyon (HCL), Lyon 69500, France; 11 Department of Paediatric Neurology Outpatient Clinic/Neonatal Paediatrics and Intensive Care, University Hospital of Rouen, Rouen 76000, France

**Keywords:** alternating hemiplegia of childhood, progression, disability, Mashlool D801N mouse, *ATP1A3*

## Abstract

Alternating hemiplegia of childhood is a rare neurodevelopmental disorder caused by *ATP1A3* mutations. Some evidence for disease progression exists, but there are few systematic analyses. Here, we evaluate alternating hemiplegia of childhood progression in humans and in the D801N knock-in alternating hemiplegia of childhood mouse, Mashlool, model. This study performed an ambidirectional (prospective and retrospective data) analysis of an alternating hemiplegia of childhood patient cohort (*n* = 42, age 10.24 ± 1.48 years) seen at one US centre. To investigate potential disease progression, we used linear mixed effects models incorporating early and subsequent visits, and Wilcoxon Signed Rank test comparing first and last visits. Potential early-life clinical predictors were determined via multivariable regression. We also compared EEG background at first encounter and at last follow-up. We then performed a retrospective confirmation study on a multicentre cohort of alternating hemiplegia of childhood patients from France (*n* = 52). To investigate disease progression in the Mashlool mouse, we performed behavioural testing on a cohort of Mashlool^-^ mice at prepubescent and adult ages (*n* = 11). Results: US patients, over time, demonstrated mild worsening of non-paroxysmal disability index scores, but not of paroxysmal disability index scores. Increasing age was a predictor of worse scores: *P* < 0.0001 for the non-paroxysmal disability index, intellectual disability scale and gross motor scores. Earliest non-paroxysmal disability index score was a predictor of last visit non-paroxysmal disability index score (*P* = 0.022), and earliest intellectual disability score was a predictor of last intellectual disability score (*P* = 0.035). More patients with EEG background slowing were noted at last follow-up as compared to initial (*P* = 0.015). Similar worsening of disease with age was also noted in the French cohort: age was a significant predictor of non-paroxysmal disability index score (*P* = 0.001) and first and last non-paroxysmal disability index score scores significantly differed (*P* = 0.002). In animal studies, adult Mashlool mice had, as compared to younger Mashlool mice, (i) worse balance beam performance; (ii) wider base of support; (iii) higher severity of seizures and resultant mortality; and (iv) no increased predisposition to hemiplegic or dystonic spells. In conclusion, (i) non-paroxysmal alternating hemiplegia of childhood manifestations show, on average over time, progression associated with severity of early-life non-paroxysmal disability and age. (ii) Progression also occurs in Mashlool mice, confirming that *ATP1A3* disease can lead to age-related worsening. (iii) Clinical findings provide a basis for counselling patients and for designing therapeutic trials. Animal findings confirm a mouse model for investigation of underlying mechanisms of disease progression, and are also consistent with known mechanisms of *ATP1A3*-related neurodegeneration.

## Graphical Abstract

**Graphical Abstract:**
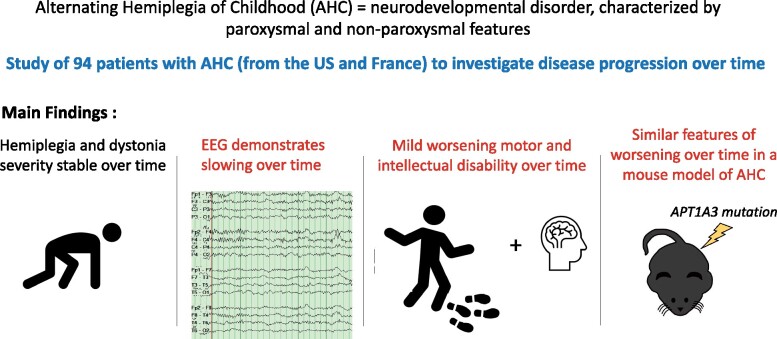

Abbreviated summary

Uchitel et al. found, in Alternating Hemiplegia of Childhood patients, that over time there is: (i) Mild worsening of non-paroxysmal neurological disability, correlating with the degree of initial (<1.5 years old) such disability, and no change in hemiplegia/dystonia related disability. (ii) More EEG slowing. (iii) Mouse model showed similar findings.

## Introduction

Alternating Hemiplegia of Childhood (AHC) is a severe paediatric neurological disorder characterized by paroxysmal events of hemiplegia and dystonia, often co-occurring with severe developmental disabilities.^1–10^ AHC has an estimated prevalence of 1/1 000 000 children, and 75% of clinically diagnosed cases are due to *de novo* mutations in the *ATP1A3* gene, most commonly, the D801N mutation.^7^ The D801N knock-in mouse model (*Mashl^+/−^*) exhibits the manifestations of the human condition.^11^^,^^12^ Clinically, AHC is diagnosed according to Aicardi’s criteria, defined as: (i) onset of symptoms before 18 months of age; (ii) paroxysmal hemiplegia; (iii) paroxysmal dystonia, nystagmus or various autonomic symptoms; (iv) episodic bilateral hemiplegia; (v) symptoms resolve with sleep; and (vi) developmental delay or other neurologic abnormalities.^4–8^^,^^10^ Previous studies have provided confounding results about the course of AHC. An initial cross-sectional study suggested that AHC follows a non-progressive course,^4^ yet several subsequent studies reported that patients may have regression (varying 2–33%, depending on the series) and even catastrophic deterioration.^4^^,^^6^^,^^13^^,^^14^ These findings suggest a progressive course in at least some patients. The aims of this study were to test the following hypotheses: (i) the severity of AHC, as determined by scores on scales of paroxysmal and non-paroxysmal disability, follows a progressive course, worsening with age. (ii) Early-life clinical variables can predict such changes. (iii) The D801N knock-in mouse model (*Mashl^+/^*^*−*^) shows age-related worsening in its AHC-like manifestations.

## Materials and methods

### Ambidirectional analysis of clinical data

#### Inclusion criteria and clinical testing

Inclusion criteria: (i) patient fulfilled the above-mentioned Aicardi’s clinical criteria,^4^^,^^5^^,^^10^^,^^15^^,^^16^ and (ii) Patient underwent two or more neurological and developmental evaluations at least 1 year apart. Forty-two consecutive AHC patients who were seen in the Duke AHC Multidisciplinary Clinic over a period of 6 years (2013–19) fulfilled these criteria. Patients underwent clinical evaluations and tests according to clinical need and to our AHC clinical pathway (includes genetic, EEG and MRI investigations, see Masoud et al.^10^). Genetic testing was performed through whole exome sequencing or through a targeted gene panel that included *ATP1A3, ATP1A2, SLC2A1, SCN1A, PRRT2, CACN1A*. Some patients had had additional panels done via Next Generation Sequencing. When gene testing was positive, it was confirmed by Sanger sequencing. For the US patients, written informed consent was obtained from all participants or guardians. All data were entered into our Duke Institutional Review Board-approved database. Similarly, all French patients were consented to be included in the prospective observational cohort, and to contribute data as appropriate to all related studies.

#### Ambidirectional analysis: maximizing the benefits of retrospective and prospective data

**Figure 1:**
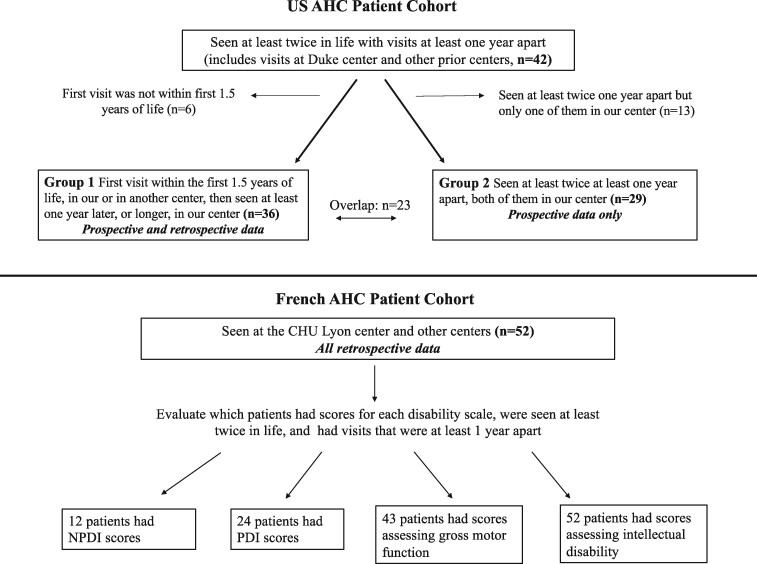
**Summary of study population.** Flowchart detailing the characteristics of each cohort. For the US cohort of AHC patients, we initially identified 42 patients who were seen at least twice in life with visits at least 1 year apart, with visits occurring either at the Duke Multidisciplinary AHC Center or other prior centres. Patients were then classified into two groups. Group 1 (*n* = 36) contains patients with ambidirectional data (prospective and retrospectively collected data), which was analysed to determine whether disease severity, according to the aforementioned disability scales, progressed over time. Group 2 (*n* = 29) contains only prospectively acquired data from patients who were followed at the Duke centre over the years of 2013–2020. Details of the French AHC data are also presented. As a reminder for the reader, NPDI assesses global neurological functioning, PDI assesses the severity, frequency and duration of hemiplegia and dystonia, GMFCS assess gross motor function, and IDS assesses intellectual disability.

For the cohort of 42 patients, some patients were seen in prior centres in addition to our centre. As such, the data that we collected consisted of both retrospectively collected data (data from prior centres) and prospective data (data from our centre). Analysis of this type of data constitutes an ambidirectional study. Ambidirectional studies, which have been used in multiple disciplines to study clinical course of disease, combine the advantages of prospective and retrospective studies while minimizing, though not necessarily completely eliminating, the disadvantages of both types of studies, by allowing for the investigation of prospectively acquired data while also being able to analyse retrospectively available data.^17–21^ Thus, the 42 patients were divided into two groups (Fig. 1) based on whether they either: (i) had their first visit within the first 1.5 years of life in another centre, then were seen at least 1 year later in our centre (ambidirectional data, Group 1), or (ii) were seen at least twice in our centre with visits at least 1 year apart (prospective data, Group 2). As stated above, the aim of this method was to ensure that all available data (retrospectively collected and prospectively collected data for Group 1) would be analysed, and also to analyse prospectively collected data separately (Group 2), because prospective data are more are generally accepted to be more reliable. Group 1 (retrospective and prospective data) included 36 patients seen during the first 18 months of life, whether in our clinic or in an outside centre, as well as later in our clinic at least 1 year after their initial visit. This 18-month cut-off was chosen because one of the Aicardi diagnostic criteria is onset of AHC before the age of 18 months. Group 2 (prospective data only) included 29 patients who were seen at least twice in our Multidisciplinary AHC Clinic with visits at least 1 year apart by the same team using the same clinical pathway. Although there is an overlap of 23 patients between the two groups, we considered the above approach, which analyses all available data using multiple methods, as the best approach to address the hypotheses of this study and to confirm its findings. AHC disability scores, used in prior studies and described below, were used to score the severity of AHC symptoms. These were ascertained as follows: For Group 1, detailed records of neurological and developmental evaluations during the first 18 months of life were reviewed to determine disability scores within this time period (scales described below). This initial encounter occurred at either a prior centre or at our centre. In addition, all subsequent encounters that were in our centre, and were at least 1 year apart, were reviewed to determine disability scale scores at minimum 1-year intervals. For Group 2, disability scale scores were determined for the first encounter in our centre and then at the time of most recent follow-up in the same centre. The first encounter for those in Group 2 did not necessarily need to occur within the first 18 months of life but had to have occurred in our centre. Two independent investigators from our group calculated disability scale scores for each patient at each of the above time points (J.U. and L.T.) and were blinded to each other’s scores. The mean of the two was used for statistical analysis. To describe how strongly these two scores resembled each other we calculated the intraclass correlation coefficient (ICC).

### Scales and measures of neurological disability

#### Paroxysmal disability index

The paroxysmal disability index (PDI) assesses the paroxysmal features of AHC (hemiplegia and dystonia, not seizures) and was developed specifically for use in patients with AHC.^4^ This index (range: 0–24 points) assesses both plegic (A) and tonic/dystonic (B) attack severity, frequency and duration: (i) Severity, number of extremities involved (1 limb = 1 point, > 1 limb = 2 points, both sides or 4 limbs = 3 points); (ii) frequency (<1 attack/year = 1 point, monthly attacks = 2 points, weekly = 3 points, daily = 4 points); (iii) duration (<1 h = 1 point, 1–6 h = 2 points, 6–12 h = 3 points, 12–24 h = 4 points, >24 h = 5 points). Higher PDI scores correspond to more severe paroxysmal hemiplegic/dystonic features of AHC. The PDI is a clinical tool developed by the European Association & Network for Research on Alternating Hemiplegia (ENRAH) consortium, in order to facilitate quantification of clinical observations, specifically for patients with AHC.^4^^,^^22^

#### Non-paroxysmal disability index

The non-paroxysmal disability index (NPDI) (range: 0–15 points) assesses global neurological impairment as defined by the sum of scores allocated to seven possible variables. NPDI scores were also determined according to Panagiotakaki et al.^4^ NPDI and PDI developed by the ENRAH consortium, in order to facilitate quantification of clinical observations, specifically for patients with AHC, and were used for this study, as in previous studies.^4^^,^^22–24^

The following modifications were made for the needs of this study: we used the Manual Ability Classification System (MACS)^25^ to assess fine motor function, the Gross Motor Function Classification System (GMFCS)^26^ to assess gross motor function and the intellectual disability scale (IDS) described previously by Katz and Lazcano-Ponce^27^ to assess for degree of intellectual impairment. These modifications were made as the original NPDI does not specify how to clinically score these features. All of these scales have been used in patients with AHC.^14^^,^^23^^,^^28^^,^^29^ The MACS and the GMFCS are rated on a scale of I–V, and the IDS ranges from none to profound (none, mild, moderate, severe and profound). In our study, a patient could obtain a maximum of 3 points for fine motor abnormalities (as determined by the MACS level: level I = 0, level II = 1, level III or IV = 2, level V = 3 points), a maximum of 3 points for gross motor (as determined by the GMFCS level: level I = 1; level II or III = 2; level IV or V = 3) and a total of 4 points for intellectual disability (none = 0, mild = 1, moderate = 2, severe = 3, profound = 4). The NPDI was, thus, defined as the sum of scores allocated to seven variables: (i) the ability to walk independently (independent walking = 0 points, walking with help = 1 point, not possible = 2 points); (ii) behavioural disorder (no = 0 points, yes = 1 point); (iii) communication disorder (no = 0 points, yes = 1 point); (iv) degree of gross motor abnormalities; (v) degree of fine motor abnormalities; (vi) presence of a movement disorder (chorea, dystonia, myoclonus, tremor and complex movement disorders: no = 0 points, yes = 1 point); and (vii) degree of intellectual disability (variables 4, 5 and 7 were quantified as described above). A higher NPDI score corresponds to more severe non-paroxysmal features of AHC.

#### Intellectual disability scale

Severity of intellectual disability was categorized as mild, moderate, severe or profound based on patients’ neurological and developmental exams classified according to the above intellectual disability assessment scale^27^ and as previously used in patients with AHC.^23^ This scale is stratified by age to account for developmental differences in different age groups. In addition to being included in the NPDI score, the degree of intellectual disability as assessed by the IDS score was also analysed separately, as a secondary outcome measure in contrast to the two primary outcome measures above, from NPDI due to its particular clinical relevance. Similar to the other scales, a higher IDS score corresponds to more severe impairments.

#### Gross Motor Function Classification Scale (GMFCS)

The GMFCS is a five-level (I–V) classification system assessing everyday gross motor abilities of individuals with cerebral palsy (CP) and other disorders, including AHC.^26^^,^^28^^,^^30^ The GMFCS measures the following age‐specific skills: (i) walking without limitations; (ii) walking with limitations; (iii) walking using a hand‐held mobility device; (iv) self‐mobility with limitations, including the use of powered mobility; and (v) transportation in a manual wheelchair. Higher GMFCS scores correspond to more severe gross motor impairments. In addition to being included in the NPDI score, the degree of gross motor disability as assessed by the GMFCS score was analysed separately, as a secondary outcome measure, from NPDI due to its particular clinical relevance.

#### Electroencephalograms

Electroencephalograms (EEGs) were assessed for background slowing for all 42 US patients. Five other consecutive US AHC patients who fulfilled the inclusion criteria and who were seen at the Duke clinic were also later included, as they were seen at our centre after initial data collection and analysis for the original cohort. We categorized the EEG into normal background or into background showing any slowing whether focal, lateralized or generalized. Accordingly, we determined if the first and last EEGs of these patients (total: *n* = 47) had any background, non-paroxysmal, slowing. First and last EEG backgrounds were then classified as normal or slow. We did not investigate paroxysmal EEG features or features of epilepsy in this study as these have been previously analysed, in detail, and published in a previous study by us.^14^

### Retrospective study of French AHC patients

In order to confirm that findings from the above investigations were not unique to one centre, we analysed data collected from a cohort of 52 AHC patients seen at multiple centres in France over a period of 35 years (1985–2020). These data were mostly collected retrospectively, although some patients were followed prospectively at the Pediatric Clinical Epileptology and Functional Neurology department, University Hospitals of Lyon. Not all 52 patients received an evaluation of the NPDI and PDI or gross motor function at two or more consecutive time points at least 1 year apart (as done for Group 1 in US patients), and as such, patients were only included in the analysis if they had at least two evaluations at least 1 year apart for NPDI, PDI, gross motor function, or intellectual disability (*n* = 12, *n* = 24, *n* = 43, *n* = 52, respectively). We note that, for the degree of gross motor function and degree of intellectual disability, these patients were not evaluated using formal evaluation scales (such as the GMFCS and the scale by Katz and Lazcano-Ponce^27^) that were used for the US patients. In addition, these patients were not all evaluated at the same centre by the same clinicians.

### Animal studies

All procedures were approved by the Duke University Institutional Animal Care and Use Committee. To determine if our established *Mashl^+/−^* mouse also shows progression, we used multiple tests to assess for AHC-related function as utilized in previous studies both in epilepsy and in AHC models.^11^^,^^12^^,^^31–33^ We performed a battery of behavioural tests on age‐matched wild-type (WT) and *Mashl^+/−^* littermates beginning at age 31 days (WT *n* = 14; *Mashl^+/−^ n* = 11; pre-pubescent period), then repeated the same tests on the same cohort beginning at age 121 days (as adults). The following behavioural tests, chosen specifically because they were previously shown by us to detect AHC-like manifestations in AHC mice,^11^^,^^12^^,^^31^ were performed: (i) balance beam, accelerating rotarod and gait analysis to assess motor coordination; (ii) grip strength to assess motor strength; (iii) novel object memory test; and (iv) open field to assess hyperactivity and impulsivity. The number of open field crosses is an accepted measure of hyperactivity, and the time spent in the centre is an accepted measure of impulsivity.^12^^,^^32^^,^^33^ In addition, cold-water testing was used to induce hemiplegia, dystonia and seizures as described previously in *Atp1a3* D801Y mutant mice.^34^ In *Mashl^+/−^* mice, these induced AHC spells begin with dystonia, followed by hemiplegia, and finally by behavioural seizure activity. WT mice do not demonstrate any occurrences of such paroxysmal behaviour. Thus, this test is useful to assess predisposition to induced AHC spells in *Mashl^+/−^* mice. The occurrence of hemiplegia and dystonia was scored as 0 = absence, 1 = presence. Seizure severity was scored according to the Racine scale for mice.^35^

### Statistical analyses

For all tests, statistical significance was determined by *P* < 0.05 and using two-tailed tests as appropriate. In cases of multiple comparisons, a Bonferroni correction was applied. Clinical data were analysed using RStudio (PBC, Boston, USA). SigmaPlot 11.0 (Systat Software, Inc., San Jose, USA) was used for animal data analysis. Data are presented as mean ± SE.

#### Investigating potential progression over time in US patients

To investigate how disability scale scores may change over time, we used linear mixed effects models.^36^ Linear mixed effects models are an extension of linear regression models. They use random effects to account for variability among individuals and allow for analysis of repeated measures over time. This type of analysis has been used in previous natural history studies of neurological disorders, including Canavan disease, Friedreich ataxia, cerebral palsy, Parkinson’s disease and Alzheimer’s disease.^36–42^ Using data from US patients in Group 1, we fit four linear mixed effects models, one for each of the following scores: NPDI, PDI, IDS and gross motor scores. Age was included as a fixed effect, and each patient was classified as a random effect to account for individual differences in disease progression. For Group 2, we used the Wilcoxon-signed rank test for paired data to compare disability index scores at first and last encounter.

#### Investigating early life predictors of later disease severity

We used multivariable regression models with data from Group 1 (all seen within the first 18 months of life) to determine potential early-life predictors of current disability. The following clinical variables were investigated as predictors: age as of last follow-up, earliest NPDI score, earliest PDI score, earliest IDS score, earliest gross motor score, sex, *ATP1A3* status (positive or negative) and presence of epilepsy. Age at last follow-up was chosen to determine whether older patients were more likely to have worse scores, while earliest NPDI, PDI, IDS and gross motor scores were chosen to determine if severity of disease in a patient during early-life (within the first 18 months) could predict later disease severity.

#### Investigating the effect of flunarizine

Flunarizine is a known and widely-used medication for the treatment of AHC. A number of studies have shown that it can reduce the severity, frequency and duration of paroxysmal attacks.^4^^,^^43–47^ A previous study by one of us reported that, whereas flunarizine reduces the above spells, the duration of its use did not correlate (via least squares linear regression), with the long-term developmental status.^2^ To determine whether the use of flunarizine over time affected the degree of developmental impairments in this current study, we investigated the correlation between change in disability index scores for patients in Group 2 to these patients’ flunarizine index using Spearman’s Rho. The flunarizine index is calculated as the duration of flunarizine treatment divided by the patient’s age.^2^ Group 2 was chosen as these patients were followed prospectively and, thus, the clinical records of their treatment with flunarizine (time on and off) are more well-documented. This analysis was considered exploratory as the number of patients, with information about the flunarizine index, was only 22.

#### EEG data

To determine whether more patients demonstrated EEG slowing at last follow-up as compared to the first encounter, we used the McNemar test.

#### Investigating potential progression over time in French patients

In the supplementary cohort of French AHC patients, linear mixed effects models were also used to investigate how disability scale scores may change over time (as performed for US patients in Group 1 above). Scores at the first encounter and the last follow-up were also compared using the Wilcoxon Signed Rank test for paired data (as performed for US patients in Group 2 above).

#### Animal data statistics

Data from *Mashl^+/−^* mice were normalized to age-matched wild-type mice, presented for each *Mashl^+/−^*mouse as ratio of the *Mashl^+/−^* mouse to the mean of wild-type (HET/WT) for each variable. In this study, as in our prior studies, data were pooled between male and female mice except for variables that showed sex differences (only gait analysis variables showed sex differences).^11^^,^^12^ A paired two-tailed Student’s *t*-test was used for normally distributed data. Data not normally distributed were analysed using the Mann–Whitney U-test. Categorical data were analysed with the Kruskal–Wallis test. Nominal data were analysed with Chi-square or Fisher Exact test as appropriate.

### Data availability

Anonymized article data are available from the corresponding author upon reasonable request.

## Results

### US cohort patient characteristics

Our cohort of 42 consecutive patients (26 females, 16 males) were 10.24 ± 1.48 (mean ± SE) years old at the time of last follow-up (range of age: 1–43 years). Frequency of hemiplegic episodes ranged from multiple times per day to once per month. Thirty-nine patients had gene testing (described above), of whom 27 (69%) were positive for *ATP1A3* mutations: 8 had the D801N mutation, 4 had the E815K mutation and 14 had other mutations. The three patients who were pending gene testing were excluded from the multivariable regression, which investigated the effect of mutation status. Further details on the individual clinical characteristics of these patients are included in Supplementary Table 1.

### Effect of age

#### Progression in non-paroxysmal features of AHC over time in Group 1

**Figure 2:**
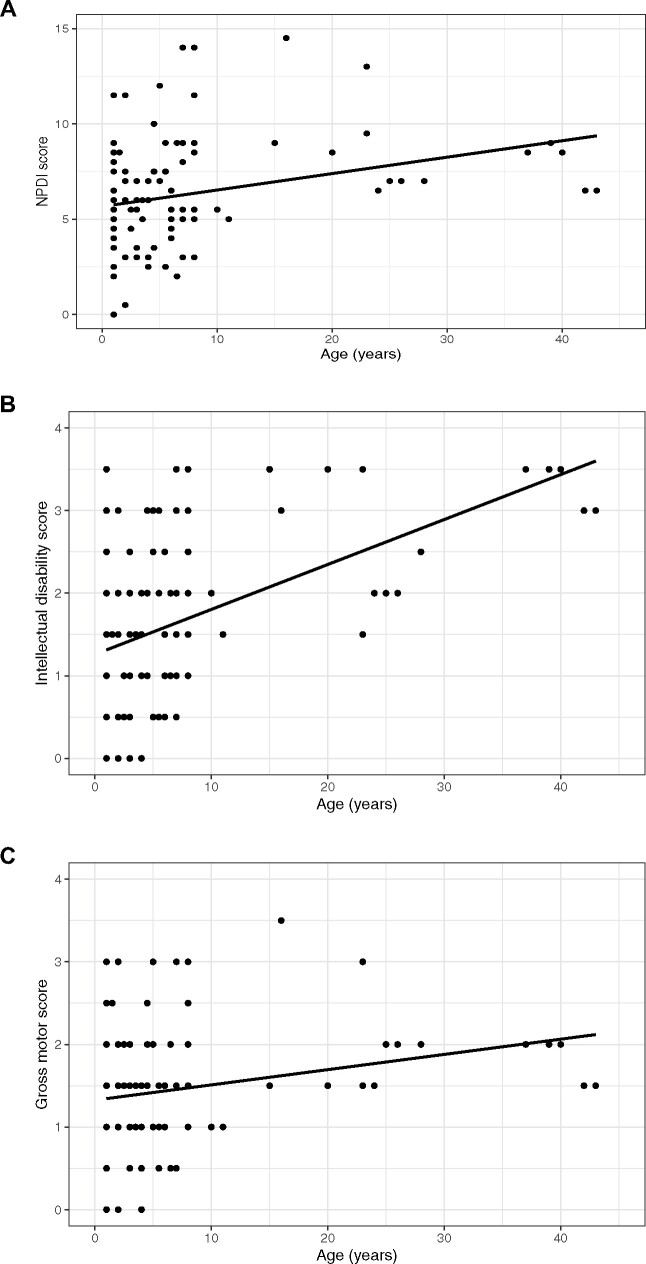
**Non-paroxysmal features of AHC worsen over time (US Group 1 data).** Age was found to be a significant predictor of non-paroxysmal disability index score, IDS score, and gross motor score in linear mixed effects modelling with patients’ increasing age associated with worse scores. This indicates that as patients get older, the non-paroxysmal features of their disease actually progress, becoming more severe over time. All scores for all encounters for each patient are plotted as age versus disability scale score. A linear regression line is shown to provide a general sense of the trend. For each of the scales, a higher score corresponds to more severe disability. However, simple linear regression was not the analysis that was used (see methods and results sections). As described in the text, we used linear mixed effects models, which allowed us to investigate group age-related changes over time. (**A**) Age versus non-paroxysmal disability index score for all patients in Group 1 for all encounters (*n* = 36, *P* < 0.0001). (**B**) Age versus Intellectual Disability Scale score for all patients in Group 1 for all encounters (*n* = 36, *P* < 0.0001). (**C**) Age versus gross motor score for all patients in Group 1 for all encounters (*n* = 36, *P* < 0.0001).

For Group 1 (retrospective and prospective data), the severity of non-paroxysmal features of AHC was found to worsen over time: age was a significant predictor of NPDI (*P* < 0.0001), gross motor (*P* < 0.0001) and IDS scores (*P* < 0.0001) in linear mixed effects models, indicating that increasing age was associated with worsening of each of these scores. In contrast, the severity of paroxysmal features of AHC was not found to worsen over time: age did not have a significant effect on PDI scores (*P* = 0.847) (Fig. 2). When the one patient who had catastrophic regression was eliminated from the group and the analysis was repeated, results were similar with significance for NPDI (*P* < 0.0001), GMFCS (*P* < 0.0001) and IDS scores (*P* < 0.0001) but not for PDI (*P* = 0.675), suggesting that catastrophic regression was not driving the observed worsening over time. The ICC values for the two AHC-specific scales were the following: NPDI, ICC = 0.457; PDI, ICC = 0.596. These values are indicative of fair reliability (0.40–0.59) (Cicchetti, 1994), and are comparable to the reliability of the NIH Toolbox Cognitive Battery in children (ICC range between 0.31 and 0.76).^48^

Additional linear mixed effects models were fit for only those patients positive for an *ATP1A3* mutation in Group 1 (*n* = 23). Again, non-paroxysmal features, but not paroxysmal features, worsened over time: advancing age was found to be a significant predictor of worse NPDI (*P* < 0.0001), gross motor (*P* < 0.0001) and IDS scores (*P* < 0.0001) but had no effect on PDI scores (*P* = 0.481). The one patient who had catastrophic regression was *ATP1A3* mutation negative and, thus, was not part of the analysis.

##### Worsened non-paroxysmal features of AHC in Group 2 after prospective follow-up

**Figure 3:**
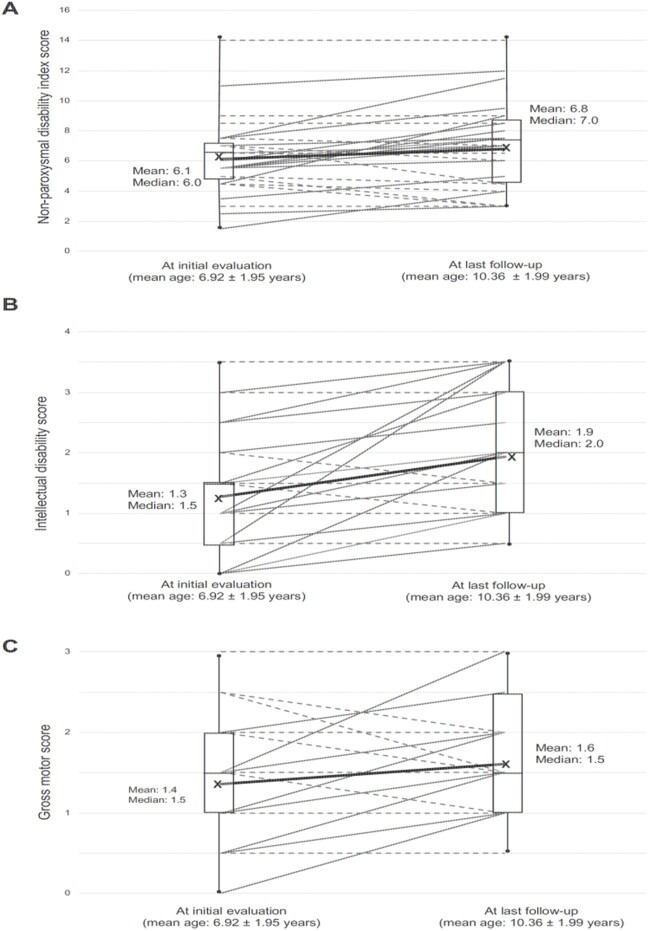
**Worsening of non-paroxysmal features of disability after prospective follow-up (US Group 2 data).** Prospectively-followed US patients also demonstrate worsening over time of non-paroxysmal features of disability. (**A**) Change in non-paroxysmal disability index scores between initial encounter in our clinic and last follow-up in our clinic (*P* = 0.042). Changes are plotted as lines between initial evaluation and last follow-up. Continuous red lines indicate worsening scores between initial encounter and most recent follow-up, hatched-green lines indicate either improving scores or no change in scores between initial encounter and last follow-up (ages: mean ± SE). Boxplots display minimum, first quartile, median, mean, third quartile and maximum values for all patients at the initial encounter on the left and at last follow-up on the right. Solid black lines connect mean values (**B**) Change in Intellectual Disability Scale Scores between initial encounter in our clinic and last follow-up in our clinic (*P* < 0.001) Same boxplot representations as for A. (**C**) Change in gross motor score between initial encounter in our clinic and last follow-up in our clinic (*P* = 0.037). Same boxplot representations as for A and B.

For Group 2, significant differences were observed between first and most recent IDS scores (*P* < 0.0001), (significant with Bonferroni correction, alpha = 0.0125) but not for NPDI scores or gross motor (*P* = 0.043, *P* = 0.037) or for PDI scores (*P* = 0.657; Fig. 3). Of note is that these comparisons were performed on the means whereby some patients’ PDI scores worsened and some improved over time with the resultant no significant differences between the means of the PDI scores. When the analysis was performed on only *ATP1A3* positive patients in Group 2 (*n* = 18), NPDI scores and IDS scores were significant (*P* = 0.009, *P* = 0.002, respectively) but gross motor and PDI scores were not (*P* = 0.119, *P* = 0.477, respectively).

##### Potential effect of flunarizine

We found no significant effect of flunarizine on the change in disability index scores after prospective follow-up for Group 2 (NPDI: *r_s_* = 0.366, *P* = 0.051; PDI: *r_s_* = −0.088, *P* = 0.650; IDS: *r_s_* = 0.058, *P* = 0.767; gross motor: *r_s_* = 0.2199, *P* = 0.255). However, given the NPDI low *P*-value, future studies are needed to further determine if there may be some effect when larger numbers of patients are included. As indicated above, this analysis was considered exploratory due to the limited number of patients that could be analysed. This prevented correcting for potential effects of possible confounding variables, such as disease severity, earliest NPDI score and age. We note that the above finding is also consistent with our prior study in which we found that the duration of flunarizine use did not correlate with the long-term developmental status.^2^

#### More abnormally slow EEG background after prospective follow-up

Fourteen patients had an initially normal EEG background that showed a slow background at later FU, 7 had a slow background on both their initial and final EEGs and 3 had a slow background on their initial EEG, but a normal final background on their final EEG. The remaining 23 patients had normal background on both initial and final EEGs. These differences were found to be significant (*P* = 0.015), indicating a predisposition to EEG background slowing with increasing age. For the 47 patients, the mean age at first EEG was 3.5 ± 4.8 years and at last EEG was 8.3 ± 6.1 years. We found that the group with EEG slowing was more likely to have higher GMFCS scores (*P* = 0.032, Mann–Whitney *U*-test), but PDI, NPDI and IDS scores did not differ (*P* > 0.2 in all comparisons). However, we consider these analyses are exploratory since our study was not planned nor powered to investigate these relationships. Of the 47 patients, 18 had epileptiform discharges in the form of focal spikes, focal sharp waves or generalized spike slow waves. We have previously reported on the details of the AHC epileptiform EEG changes and of epilepsy in a recent article^14^ and, thus, did not duplicate those results in this article.

### Early-life predictors of later disability outcome

Since NPDI scores and IDS scores demonstrated worsening with age, we investigated if early life clinical variables could predict later NPDI and IDS scores. As seen in Table 1, age as of last follow-up was a significant predictor of both current NPDI and current IDS score (*P* = 0.030, *P* = 0.0002), earliest IDS was a significant predictor of IDS score at last FU (*P* = 0.035) and earliest NDPI score was a significant predictor of NPDI score at last FU (*P* = 0.022). We also investigated the potential effect of status epilepticus (SE), present in 11/42 patients, on NPDI as of last FU by including SE as a variable in our multivariable regression (Table 1). SE was not significant (*P* = 0.9542) and also lowered the model’s *R*^2^ value, suggesting that it was inappropriate to include, and as such was removed. These findings suggest that the regression we observed in our 42 patients was independent of SE. This, however, does not rule out that, in certain individuals, regression does occur after SE, as mentioned above in one of our patients and as noted previously.^14^

**Table 1:** Results of multivariable regression for Group 1 for NPDI and IDS

Outcome variable	Predictors						
	Age as of last FU	Earliest NPDI Score	Earliest IDS Score	Earliest gross motor score	Presence of epilepsy	Mutation Positive	Sex
NPDI score at last FU (*P*-value)	0.030^*^	0.022^*^	0.267	0.358	0.933	0.588	0.313
IDS score at last FU (*P*-value)	0.0002^*^	0.152	0.035^*^	0.287	0.142	0.548	0.804

*n* = 33, all patients tested for an *ATP1A3* mutation (3 not tested in Group 1). NPDI *R*^2^ = 0.637, IDS *R*^2^ = 0.743. As a reminder for the reader, the NPDI assess non-paroxysmal features of AHC and global neurological function. The IDS score assesses a patient’s degree of intellectual disability. The gross motor score assesses gross motor disability using the GMFCS. For all of these scales, a higher score indicates more severe disability.

FU, follow-up; IDS, intellectual disability scale; NPDI, non-paroxysmal disability index; PDI, paroxysmal disability index.

* *P*-value (alpha  < 0.05).

### French cohort results

In the 52 patients in the French cohort (26 females, 26 males), the mean age at initial evaluation was 7.38 ± 7.58 years (range: 2–23 years) and mean age at last follow-up was 17.33 ± 8.21 years (range: 3–39 years). Forty-one patients were positive for an *ATP1A3* mutation (18 D801N, 6 E815K, 4 G947R, 13 other), 5 were mutation negative and 6 were mutation unknown/not yet tested. In these French patients, similar to US patients, the severity of non-paroxysmal AHC features worsened over time: age was a significant predictor for NPDI scores (*P* = 0.001) in linear mixed effects models. The degree of intellectual disability and the degree of gross motor impairment were not found to show worsening over time (*P* = 0.120, *P* = 0.074 for age). As found in US patients, the severity of paroxysmal features did not worsen over time (*P* = 0.317 for age). Comparison of first and last scores for each available index revealed that NPDI scores and degree of intellectual disability significantly differed between first and last encounter (*P* = 0.002, *P* = 0.008, significant with Bonferroni correction for 4 comparisons alpha = 0.0125), but PDI and degree of gross motor disability did not (*P* = 0.648, *P* = 0.322). Further details on the individual clinical characteristics of these patients are included in Supplementary Table 2.

### Animal study results

#### Adult *Mashl*^+/-^ mice had greater degrees of motor impairments than young *Mashl*^+/-^ mice

**Figure 4:**
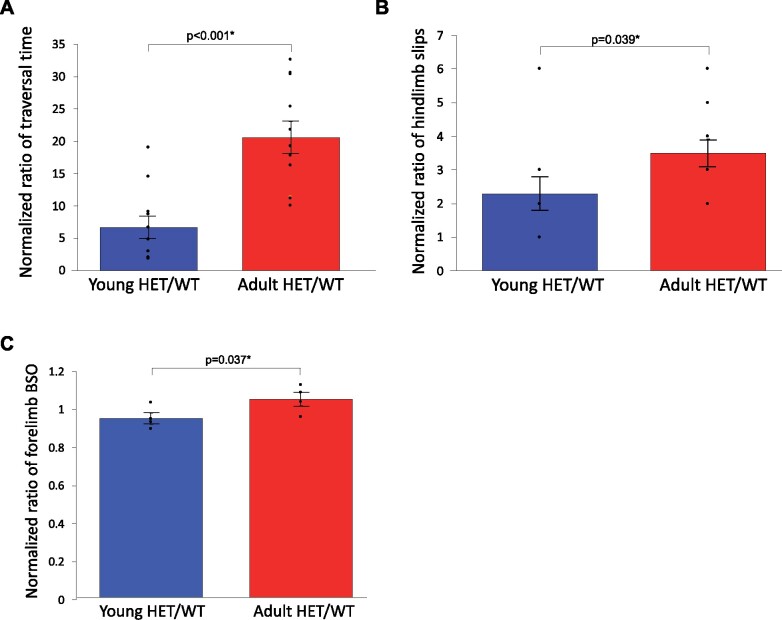
**Animal data: Adult D801N *Mashl^+/−^* mice have worse motor performance than young D801N *Mashl^+/−^* mice.** Balance beam testing was performed at 31 days of age and repeated at 118 days of age. Gait testing was performed at 32 days of age and repeated at 119 days of age. Wild-type *n* = 14; *Mashl^+/−^ n* = 11. All data were normalized to age-matched wild-type counterparts, presented as a comparison of young HET/WT (*Mashl^+/−^/*wild-type) ratios and adult HET/WT ratios (each mutant mouse data value was divided by the mean of the age matched wild-type value of the same variable to generate the HET/WT ratio for that mouse and the ratios of the two different age groups were compared). Bars indicate standard error. Dots indicate individual mouse data. (**A**) Adult *Mashl^+/−^* mice demonstrated significantly increased time to traverse the beam when compared to young *Mashl^+/−^* mice (*P* < 0.001, *t* = −6.967, two-tailed paired *t*-test, young HET/WT *N* = 11, adult HET/WT *N* = 11). (**B**) Adult *Mashl^+/−^* mice demonstrated significantly increased hindlimb slip scores when compared to young *Mashl^+/−^* mice (*P* = 0.039, H = 4.263, Kruskal–Wallis one-way ANOVA on Ranks, young HET/WT *N* = 11, adult HET/WT *N* = 11). Hindlimb slip scores were assigned as follows: 1 = 0–5 slips, 2 = 5–50 slips. 3 = 50–100 slips. 4 = 100–150 slips. 5 = 150–200 slips. 6 = 200-250 slips. A hindlimb slip was defined as a slip, rotation, or fall off of the beam. (**C**) Adult female *Mashl^+/−^* mice demonstrated significantly increased forelimb base of support (BSO) when compared to young female *Mashl^+/−^* mice (*P* = 0.037, *t* = −3.595, two-tailed paired *t*-test, young HET/WT *N* = 4, adult HET/WT *N* = 4).

The following behavioural tests were statistically significant: (i) Adult *Mashl^+/^*^*−*^ mice demonstrated longer beam traversal times (*P* < 0.001; young HET/WT = 6.7 ± 1.7; adult HET/WT = 20.6 ± 2.5). (ii) Adult *Mashl^+/^*^*−*^ mice had increased occurrences of hindlimb slips while performing the balance beam test (*P* = 0.039; 2.3 ± 0.5; 3.5 ± 0.4) (Fig. 4A and B). (iii) Adult female *Mashl^+/−^* mice demonstrated an increased forelimb base of support (BSO) in comparison to their young female *Mashl^+/-^* counterparts (*P* = 0.037; young HET/WT = 1.0 ± 0.06; adult HET/WT = 1.1 ± 0.07, Fig. 4C).

There were no significant differences in the following variables between the young and adult age mice: rotarod, open field, grip strength, novel object and other gait analysis variables (Table 2, see also Supplementary Table 3). When corrected with the modified Holm-Bonferroni statistical analysis, only beam traversal time retained significance (Holm-Bonferroni *P* = 0.003 which is higher than the *P* < 0.001 of transversal time). In this study as in our prior studies,^11^^,^^12^ we observed mice to have motor seizures, dystonia, hemiplegias and motor abnormalities as early as 15 days old and that these manifestations are very well established by the fourth week of life.

**Table 2:** Behavioural tests results for young and adult *Mashl^+/−^* mice

**Behavioral test**	*P*-value	Ratio of comparison	Young mean	Young SD	Adult mean	Adult SD
Rotarod (s)	0.943	HET/WT	0.445	0.361	0.432	0.359
Open field # of crosses	0.351	HET/WT	3.140	2.998	2.094	1.923
Time spent in centre of open field (s)	0.245	HET/WT	2.247	2.769	5.441	7.543
Forelimb grip strength (N)	0.168	HET/WT	0.752	0.146	0.874	0.240
Hindlimb grip strength (N)	0.063	HET/WT	0.783	0.142	0.962	0.248
Gait forelimb stride length (cm)	0.231	Male HET/WT	0.645	0.103	0.723	0.024
	0.357	Female HET/WT	0.615	0.172	0.788	0.241
Gait hindlimb stride length (cm)	0.204	Male HET/WT	0.663	0.100	0.743	0.044
	0.096	Female HET/WT	0.717	0.059	0.880	0.146
Gait forelimb BSO (cm)	0.536	Male HET/WT	0.993	0.123	1.023	0.171
Gait hindlimb BSO (cm)	0.073	Male HET/WT	1.118	0.037	0.966	0.110
	0.250	Female HET/WT	0.998	0.166	1.117	0.059
Novel object (s)	0.453	HET/WT	1.734	2.428	0.311	4.934

Please note that the units are included to indicate the units of the measurements before the ratios (which does not have units) were calculated and that the statistical comparisons were between ratios of HET/WT of each variable of young and adult mice (as per methods section).

BSO, base of support.

#### Increased seizure severity and seizure-induced mortality in adult *Mashl*^+/-^ mice

**Figure 5:**
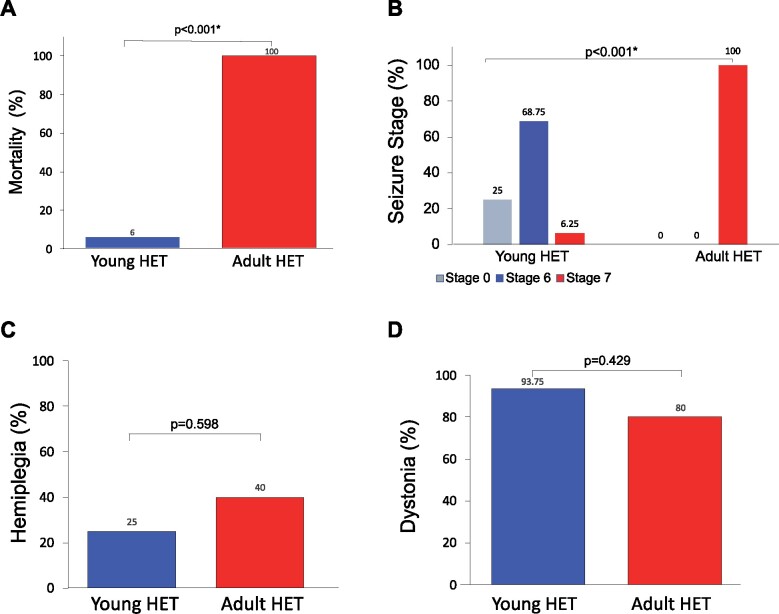
**Animal data: Adult D801N *Mashl^+/−^* mice are more predisposed to seizures than young D801N *Mashl^+/−^* mice. Comparison of cold-water test results between young and adult D801N *Mashl^+/−^* mice. Bars indicate standard error.** (**A**) Adult *Mashl^+/−^* mice demonstrated significantly increased mortality due to cold-water-induced seizures than young *Mashl^+/−^* mice (*P* < 0.001, Fisher’s exact analysis, young *Mashl^+/−^* mice *N* = 16, adult *Mashl^+/−^* mice *N* = 5). One of the 16 young mice and all the 5 adult mice died due to cold-water induced seizures. (**B**) Adult *Mashl^+/−^* mice demonstrated significantly increased severity of seizures after cold-water testing than young *Mashl^+/−^* mice (*P* < 0.001, Fisher’s exact analysis, young *Mashl^+/−^* mice *N* = 16, adult *Mashl^+/−^* mice *N* = 5). In the young *Mashl^+/−^* mice, 4 mice had stage 0 (no seizures), none had stages 1–5 seizures, 11 had stage 6 seizures, and 1 had a stage 7 seizure. In adult *Mashl^+/−^* mice, all 5 had stage 7 seizures. (**C**) There were no statistically significant differences between occurrences of hemiplegia in young and adult *Mashl^+/−^* mice (*P* = 0.598, Fisher’s exact analysis, young *Mashl^+/−^* mice *N* = 16, adult *Mashl^+/−^* mice *N* = 5). Four out 16 young *Mashl^+/−^* mice and 2 out of 5 adult *Mashl^+/−^* mice manifested cold-water induced hemiplegia. (**D**) The occurrence of dystonia after cold-water testing was not statistically significant between young and adult *Mashl^+/−^* mice (*P* = 0.429, Fisher’s exact analysis, young *Mashl^+/−^* mice *N* = 16, adult *Mashl^+/−^* mice *N* = 5). 15 out 16 young *Mashl^+/−^* mice and 4 out of 5 adult *Mashl^+/−^* mice manifested cold-water induced dystonia.

To compare seizure predisposition of adult and young *Mashl^+/−^* mice, we compared a group of young mice (*n* = 16, age P35) and another group of adult mice (*n* = 5, age P114–166) using the cold-water induction test. This resulted in more severe seizures (*P* < 0.001) and a 100% mortality rate in adult *Mashl^+/−^* mice (*P* < 0.001, ratio dead/survived for young: 0.063 ± 0.1, for adult: 1.000 ± 0.0) (Fig. 5A and B). Furthermore, seizures in adult *Mashl^+/−^* mice were shortened as death occurred soon after the seizures started (*P* = 0.037; 956.1 ± 226.8 s; 423.2 ± 61.8 s).

#### Occurrences of hemiplegia and dystonia did not differ between young and adult *Mashl*^+/-^ mice

Dystonia and hemiplegia spells were induced by the above cold-water test. When such spells occurred, dystonia and/or hemiplegia always preceded the occurrence of the seizures. There was no significant difference in the percent occurrence of cold-water induced hemiplegia or dystonia between young and adult *Mashl^+/^^−^* mice (*P* = 0.598; *P* = 0.429, respectively; Fig. 5C and D). Supplementary Figure 1 shows illustrations of the EEG findings during baseline, and during, post-cold-water induced, hemiplegia (lower amplitude background), dystonia (lower amplitude background) and epileptic seizures (electrographic seizures).

## Discussion

### Significance of findings

In this study, we conducted a longitudinal analysis of disease course in AHC in an ambidirectional and prospective study. In both the US and French cohorts, we found an effect of age on non-paroxysmal, but not on paroxysmal, disabilities over the disease course. Our methods are well suited for future larger multicentre studies on the natural history of AHC and on therapeutic interventions, because of the following: (i) linear mixed effect models have been successfully used for the purpose of evaluating therapies and clinical course of neurological disorders^36–42^; (ii) AHC disease severity is variable between patients; and (iii) AHC is a very rare disease. Our approach and findings render the successful performance of clinical trials, with the benefit of the prognostic predictors we identified and with a better understanding of the clinical course, a more achievable goal.

When comparing the US and French cohorts, we found consistent results describing mild worsening of non-paroxysmal, but not paroxysmal, features of AHC (as per results from linear mixed effects models and the Wilcoxon test). We note that the worsening of intellectual disability and degree of gross motor impairments observed in the US patients was not observed in the French patients. However, in the French patients, these two features were not scored using formal scales (GMFCS and IDS scales) as was the case for the US cohort. For the French cohort, patients’ gross motor function and intellectual disability severity were assessed using an ordinal scale based on the clinical impression of the clinician evaluating the patient at the time. In addition, we note that the degree of intellectual disability and of gross motor impairments were only secondary outcome measures, whereas the NPDI and PDI scale scores are primary, comprehensive outcome measures. Moreover, both of these cohorts had substantial sample sizes (42 for US and 52 for French), which is often difficult to achieve in studies of rare disorders.

For both sub-groups of the US cohort (Groups 1 and 2), worsened non-paroxysmal features and NPDI score were observed. We note that even though there is a 23 patient overlap between Groups 1 and 2, our method allowed the analysis of both ambidirectional and prospective data, each with the appropriate statistical methods. Additionally, the French patients also demonstrated worsening over time in NPDI scores, supporting that this finding is not unique to one group or cohort.

We emphasize here that changes noted in non-paroxysmal disability were relatively mild. This has two implications. First, cross-sectional studies may have difficulty detecting such mild changes. Second, this type of change with age is consistent with children who may still be gaining milestones, but at a much slower rate as compared to their typically developing peers, such that they demonstrate relative worsening without necessarily having any regression. We recognize that there may be variability in care between different countries, but if this exists, then it supports that our findings are part of the disease biology and not dependent on the type of care.

Our findings indicate early life (within the first 18 months of life) non-paroxysmal disability scores (specifically, NDPI and IDS) can help predict later life scores. This should be helpful in counselling families about long-term prognosis.

### Comparisons with prior literature

Our above observations show similarities and some differences with two previous cross-sectional studies. Specifically, a large study with 157 European patients, which included many of the patients in the French cohort of our article, showed no progression of paroxysmal disability, similar to what we observe here.^4^ Unlike our observations on the NPDI, that study did not detect an effect of age on non-paroxysmal disability. However, we note that the methods used in the current article are more robust: we used statistics that allowed us to investigate changes over time at the individual and group levels, whereas the prior article used approach that grouped individuals according to age before analysis. Another difference may be related to the cross-sectional and multicentre, multinational nature of that study. First, younger patients with more severe disability could have been preferentially referred to centres participating in that study. Second, multicentre multinational studies may have increased variability resulting from assessments being performed in different facilities across several countries. This increased variability may make it more difficult to detect relatively mild age-related changes, such as what we observed in the current article. Our findings also differ from those of our prior cross-sectional study, which found that age did not correlate with GMFCS scores.^28^ However, that latter study had only 17 patients and was, thus, limited in its ability to detect effects of age.

On the other hand, our results are consistent with multiple other studies showing that a variable percentage of AHC patients, 2–33%, depending on the series, have worsening neurological disability.^4^^,^^6^^,^^13^^,^^14^ These other studies reported motor and or cognitive regression following severe hemiplegic spells, fever, or status epilepticus without regaining of prior lost function in various percentages of AHC patients (3/132 patients, 2%; 10/33, 30%; 2/51, 4%; and 3/9, 33%, respectively).^4^^,^^6^^,^^13^^,^^14^ Another study found irreversible, severe motor and intellectual deterioration in 7/14 AHC patients (50%), accompanied by progressive cerebral and cerebellar atrophy.^49^ In the latter study, however, patients appear to have been specifically selected for their severe AHC and, thus, its findings are not necessarily applicable to all other AHC patients.

Our findings are novel because we observed, overall, a slow worsening in non-paroxysmal disability, rather than just sudden regression after a catastrophic event. This result remained significant, even after the one patient who had obvious catastrophic regression was removed from the analysis of our cohort. These findings in AHC are similar to the progression of mitochondrial disease, in which not only catastrophic regression, but also slow worsening in neurological disability over time, can occur.^50^

We found that later EEGs were more likely to show slowing. The slowing of the EEG background could, thus, prove to be a useful biomarker in future clinical trials in AHC treatment. Currently, only MRI shows promise as a potential biomarker.^49^^,^^51^^,^^52^ Additionally, in our prior article,^14^ we found that 37% of AHC patients with epilepsy and epileptiform activity initially had a normal EEG that later became epileptiform (mean ± SE of lag time between EEGs: 3.53 ± 4.65 years). This suggests that, like non-paroxysmal features of AHC, EEG features may also worsen over time. Future studies should consider both non-paroxysmal features and EEG slowing as biomarkers of disease progression.

Prior studies have demonstrated that the severity of AHC manifestations is affected by the type of *ATP1A3* mutation present. Specifically, patients with E815K mutations demonstrate the most severe intellectual and motor disability, when compared to the D801N and G947R mutations.^5^^,^^13^^,^^53^^,^^54^ Here, the number of patients did not allow for a genotype–phenotype correlation for effects of age, but this may be investigated in future studies.

### Mouse model findings

Our findings in the mouse model have two implications. First, they further confirm that *ATP1A3*-related disease can lead to progression, even in the absence of catastrophic regression, further supporting our clinical observations. Second, our model may be used in future investigations of potential underlying mechanisms. Whereas these mechanisms may, potentially, be related to a predisposition of the brain carrying AHC-type mutations to increased excitotoxicity^11^^,^^12^^,^^31^^,^^55^^,^^56^ or to impaired neurogenesis or synaptogenesis,^57–59^ there are many variables that may interfere with long-term prognosis. These include the pharmacological control of seizures, the long-term effects of treatments and the standards of care and assistance. These variables should be taken into account and addressed in future studies.

### Limitations, strengths and conclusions

Patients in this study may have been specifically referred to us because of more severe disease. Nonetheless, the neurological manifestations of patients we studied are comparable to those in other series described in Europe, USA and China, and appear to be less severe than a Japanese patient cohort.^2^^,^^4^^,^^5^^,^^9^^,^^13^^,^^14^^,^^53^^,^^54^ Our study had a relatively small number of patients. Group 1 data during the earliest encounter were partly retrospective. Ambidirectional analysis may raise biases with regards to retrospective data collection and inter-examiner differences in clinical evaluation. However, the concordance between the results of US and French patient cohorts and between the human and animal data strongly supports our conclusions. Regarding potential bias from centre to centre, we would like to elucidate that we analysed both cohorts separately and found consistent results which supports that our findings are not dependent on potential biases that may exist in one centre or another. In addition, the French patients underwent the same ENRAH structured questionnaire irrespective of which centre they came from, thus, limiting any potential biases. We would like also to point out that our results are applicable to the age groups we studied (mean age at last follow-up in the USA cohort was 14.00 ± 9.55), and not beyond, particularly that we only had in the US cohort seven patients older than 18 years at age of last follow-up (their mean age: 29.0 ± 7.1, range 20–43 years old). In the French cohort mean age at last follow-up was 17.33 ± 8.21 years (range: 3–39 years) with only 23 patients older than 18 years of age at time of last follow-up (their mean age: 26.6 ± 5.3 years, range 19–39 years old). One potential limitation to our mouse data is that the adult mice were previously manipulated as part of their testing performed during prepubescence, and this could have predisposed to increased risk of stress induced neuronal injury. However, we believe our results still support age-related progression for the following reasons: (i) AHC patients also manifest hemiplegia, dystonia and seizures in response to stressors, such as exposure to water, extremes of temperature, fatigue, emotional stress and infection. (ii) We also compared balance beam performance between naïve adult *Mashl^+/^*^−^ mice who had not undergone prior cold-water testing and young *Mashl^+/^*^−^ mice. These naïve, not previously manipulated, adult mice similarly demonstrated poorer balance beam performance when compared to the young (data not shown). Another consideration is that dystonia and hemiplegia spells were provoked by cold-water induction rather than spontaneously. However, it is important to note that patients with AHC do manifest these paroxysmal behaviours in response to triggers, as noted above.^2^^,^^3^^,^^9^ Furthermore, our prior studies have demonstrated that paroxysmal spells induced by cold-water are responsive to flunarizine,^31^ as are hemiplegia spells in patients, confirming that these induced paroxysmal behaviours in mice are indeed reflective of those found in AHC patients.

Despite these limitations, our study has the advantages of an ambidirectional study, and of using multiple types of analyses across patient cohorts, and between humans and mouse models, that led to consistent conclusions. This consistency supports the use of the same methods in future larger multicentre studies of the natural history and therapeutic interventions of AHC. Given the evolving definition of the AHC syndrome and the prospects for novel gene therapy, our findings also support future larger studies of AHC to further refine the current knowledge of the course of AHC and of any other additional potential predictors of outcome over longer periods of time.^60–62^

## Supplementary material

Supplementary material is available at *Brain Communications* online.

## Supplementary Material

fcab128_Supplementary_Data

## Appendix

**Participating members of the Duke AHC Research Group:** April Boggs, Melanie Bonner, Michael Carboni, Courtney Elliot, Cecilia Fernandes, Kelly Gordon, Matthew Khayata, Amanda Hall, Ashley James, Joan Jasien, Sujay Kansagra, Dwight Koeberl, Andrew Landstrom, Kara Lardinosis, Peter Malinosky, Gary Maslow, Melissa McLean, Melissa Minton, Richard J. Noel, Aruna Rikhi, and Mary K Rogers Boruta in addition to the following coauthors: Julie Uchitel, Keri Wallace, Linh Tran, Arsen Hunanyan, Lyndsey Prange, Joan Jasien, Laura Caligiuri, Milton Pratt, Blaire Rikard.

**Participating Members of the French AHC Consortium:** Isabelle An-Gourfinkel, Thierry Billette de Villemeur, Marie-Anne Barthez, Afaf Benitto, Philippe Bensaid, Marie Bourgeois, Denys Chaigne, Marie-Pierre Chaunu, Marie-Anne Cournelle, Anne De St Martin, Beatrice Deny, Isabelle Desguerre, Vincent Des Portes, Diane Doummar, Anne Dusser, Cyril Gitiaux, Isabelle Godet Kiesel, Marie Hully, Marie Husson, Christine Ioos, Anna Kaminska, Cécile Laroche, Gaetan Lesca, Eric Le Galloudec, Laurent Magy, Cécile Marchal, Mathieu Milh, Jacques Motte, Marie-Laure Moutard, Sylvia Napuri, Marie Cécile Nassogne (Belgium), Sophie Nicole, Jean-Michel Pedespan, Marie-José Penniello-Valette, Daniel Quillerou, François Rivier, Clotilde Rivier, Agathe Roubertie, Louis Vallée, Catherine Vanhulle, Laurent Vercueil in addition to the following co-authors Maria T. Papadopoulou, Isabella Cocco, Rebecca Moré, Alexis Arzimanoglou, Eleni Panagiotakaki.

## Abbreviations

AHC =: alternating hemiplegia of childhood
ENARH =: European Association & Network for Research on Alternating Hemiplegia
GMFCS =: Gross Motor Function Classification System
ICC =: intraclass correlation coefficient
IDS =: Intellectual Disability Scale
MACS =: Manual Ability Classification System
NPDI =: non-paroxysmal disability index
PDI =: Paroxysmal Disability Index
WT =: wild-type
