# Supplementary material for: Alternating hemiplegia of childhood: evolution over time and mouse model corroboration
Source: Brain Commun. 2021 Jun 4;3(3):fcab128. doi: 10.1093/braincomms/fcab128 (PMC8361420; doi:10.1093/braincomms/fcab128)
Supplement: fcab128_Supplementary_Data [file fcab128_supplementary_data.docx]

**Supplementary Table 1. Characteristics of US AHC Patient Cohort**

| **Patient** | **AHC onset (months)** | **Age at earliest encounter (Group 1)**  **(years)** | **Age first seen at Duke (Group 2) (years)** | **Age as of last follow-up (years)** | **Total years seen at Duke center (Group 2 only)**  **(prospectively followed)** | **Total years seen at all centers, starting with earliest encounter (all patients** | **Presence of Epilepsy** | **Occurrence of SE** | **ATP1A3 status** | **Group (1,2, or both)** |
| --- | --- | --- | --- | --- | --- | --- | --- | --- | --- | --- |
| 1 | 0 |  | 16.00 | 26.00 | 10.00 | 11.00 | Yes | Yes | D810N | 2 |
| 2 | 3 |  | 5.00 | 8.00 | 3.00 | 3.00 | Yes | Yes | E815K | 2 |
| 3 | 1.5 | 1.50 |  | 8.00 |  | 6.50 | Yes | Yes | E815K | 1 |
| 4 | 12 | 0.50 | 1.50 | 8.00 | 6.50 | 7.50 | Yes | Yes | D810N | 1,2 |
| 5 | 0 |  | 7.00 | 11.00 | 4.00 | 4.00 | Yes | Yes | D801N | 2 |
| 6 | 10 |  | 5.00 | 12.00 | 7.00 | 7.00 | Yes | No | E815K | 2 |
| 7 | 3 | 0.25 | 24.00 | 28.00 | 4.00 | 27.75 | Yes | No | G775C | 1,2 |
| 8 | 0 | 0.08 | 1.50 | 8.00 | 6.50 | 7.92 | Yes | No | Not yet tested | 1,2 |
| 9 | 7 | 0.58 | 4.00 | 8.00 | 4.00 | 7.42 | Yes | No | Negative | 1,2 |
| 10 | 9 | 1.17 | 3.00 | 7.00 | 4.00 | 5.83 | Yes | Yes | Negative | 1,2 |
| 11 | 0.5 |  | 4.00 | 6.50 | 2.50 | 2.50 | Yes | No | Negative | 2 |
| 12 | 3 | 0.42 |  | 16.00 |  | 15.58 | Yes | Yes | Negative | 1 |
| 13 | 0 | 0.08 |  | 5.50 |  | 5.42 | Yes | Yes | D923Y | 1 |
| 14 | 0 | 2.00 | 2.00 | 6.00 | 4.00 | 4.00 | Yes | No | Negative | 2 |
| 15 | 4.5 | 0.38 | 37.00 | 40.00 | 3.00 | 35.49 | Yes | No | Negative | 1,2 |
| 16 | 0.5 | 0.50 |  | 23.00 |  | 22.50 | Yes | Yes | Y768H | 1 |
| 17 | 12 | 0.50 | 1.50 | 4.00 | 2.50 | 3.50 | Yes | No | Negative | 1,2 |
| 18 | 0 | 1.00 | 6.00 | 7.00 | 1.00 | 6.00 | Yes | No | Negative | 1,2 |
| 19 | 0.5 | 0.33 | 15.00 | 20.00 | 5.00 | 19.67 | Yes | No | C596Y | 1,2 |
| 20 | 3 | 0.25 | 3.00 | 4.00 | 1.00 | 3.75 | Yes | No | G775C | 1,2 |
| 21 |  | 0.67 | 1.50 | 8.00 | 6.50 | 7.33 | Yes | Yes | L326R | 1,2 |
| 22 | 3 | 0.08 |  | 5.00 |  | 4.92 | Yes | No | G89D | 1 |
| 23 | 3 | 1.00 | 7.00 | 8.00 |  | 7.00 | Yes | No | G89D | 1,2 |
| 24 | 0.5 | 0.50 |  | 8.00 |  | 7.50 | Yes | Yes | Negative | 1 |
| 25 | 15 | 1.50 |  | 6.00 |  | 4.50 | Yes | No | Negative | 1 |
| 26 | 6 | 1.00 | 42.00 | 43.00 |  | 42.00 | Yes | No | D801N | 1,2 |
| 27 | 0 | 0.83 | 0.83 | 4.50 |  | 3.67 | No | No | Q851R | 1,2 |
| 28 | 0 | 0.83 | 0.83 | 4.50 |  | 3.67 | No | No | Negative | 1,2 |
| 29 | 2.5 | 1.50 | 1.50 | 7.00 |  | 5.50 | No | No | E815K | 1,2 |
| 30 | 24 | 0.25 | 1.50 | 4.00 |  | 3.75 | No | No | D801N | 1,2 |
| 31 | 0 | 0.08 | 1.50 | 3.00 |  | 2.92 | No | No | D801N | 1,2 |
| 32 | 0 | 0.92 |  | 11.00 |  | 10.08 | No | No | G947R | 1 |
| 33 | 2 | 0.08 | 5.00 | 6.00 |  | 5.92 | No | No | Negative | 1,2 |
| 34 | 1 | 0.25 | 0.25 | 2.00 |  | 1.75 | No | No | A333T | 1,2 |
| 35 | 3 | 0.25 | 1.50 | 3.00 |  | 2.75 | No | No | D801N | 1,2 |
| 36 | 0 | 0.58 | 0.58 | 2.00 |  | 1.42 | No | No | D810N | 1,2 |
| 37 | 4 | 0.13 |  | 10.00 |  | 9.88 | No | No | Not yet tested | 1 |
| 38 | 0 | 1.25 | 1.25 | 2.00 |  | 0.75 | No | No | E815K | 1,2 |
| 39 |  | 1.50 |  | 23.00 |  | 21.50 | No | No | R756H | 1 |
| 40 | 3 | 0.25 |  | 8.00 |  | 7.75 | No | No | G89D | 1 |
| 41 | 3 | 0.25 |  | 4.00 |  | 3.75 | No | No | Not yet tested | 1 |
| 42 | 8 | 0.67 |  | 2.00 |  | 1.33 | No | No | L839P | 1 |
| Mean ±  SE OR count | 3.70±0.80 | 0.61 ± 0.08 | 6.92±1.95 years | 10.24±1.48 | 3.44 ± 0.42 | 8.81 ± 1.45 | 26 with epilepsy, 16 no epilepsy | 11 SE, 31 no SE | 27 *+*, 12 -, 3 not yet tested | 36 in Group 1, 29 in Group 2 |
| range | 1-43 years | 0.08-1.5 | 10 months-42 years | 2-43 years | 1-10 years | 1-42 years |  |  |  |  |

**Supplementary Table 2: Characteristics of French AHC Patient Cohort**

| **Patient** | **AHC at onset (months)** | **Age at earliest encounter at any center (years)** | **Age first seen at the inclusion in the cohort (years)** | **Age of last evaluation for the purpose of the cohort (years)** | **Age as of last follow-up at any center (years)** | **Total years seen at all centers,  starting with earliest encounter** | **Presence of Epilepsy** | **Occurrence of SE** | **ATP1A3 status** |
| --- | --- | --- | --- | --- | --- | --- | --- | --- | --- |
| 1 | 7 | 2 | 6 | 11 | 15 | 13 | No | No | Negative |
| 2 | 1 | 1 | 8 | 19 | 20 | 19 | Yes | No | D801N |
| 3 | 1 | 0 | 10 | 15 | 21 | 21 | No | No | G947R |
| 4 | 9 | 0 | 10 | 21 | 21 | 21 | Yes | Yes | E815K |
| 5 | 1 | 1 | 10 | 19 | 19 | 18 | Yes | Yes | D801N |
| 6 | 2 | 2 | 10 | 33 | 39 | 37 | No | No | G947R |
| 7 | 1 | 1 | 10 | 24 | 24 | 23 | Yes | Yes | S811P |
| 8 | 4 | 0 | 10 | 31 | 33 | 33 | Yes | Yes | E815K |
| 9 | 1 | 0 | 4 | 15 | 17 | 17 | Yes | No | S137Y |
| 10 | 3 | 1 | 9 | 20 | 21 | 20 | No | No | Negative |
| 11 | 1 | 0 | 3 | 11 | 15 | 15 | Yes | Yes | D801N |
| 12 | 21 | 1 | 10 | 28 | 28 | 27 | No | No | D801V |
| 13 | 17 | 1 | 7 | 14 | 17 | 16 | Yes | Yes | Negative |
| 14 | 9 | 3 | 10 | 33 | 33 | 30 | Yes | No | D801N |
| 15 | 1 | 1 | 10 | 21 | 21 | 20 | Yes | Yes | D801N |
| 16 | 1 | 0 | 7 | 12 | 20 | 20 | Yes | Yes | E815K |
| 17 | 4 | 0 | 3 | 8 | 8 | 8 | No | No | D801N |
| 18 | 1 | 8 | 10 | 28 | 30 | 22 | No | No | Negative |
| 19 | 2 | 1 | 8 | 20 | 20 | 19 | No | No | T804I |
| 20 | 3 | 0 | 10 | 16 | 16 | 16 | Yes | No | D801N |
| 21 | 14 | 15 | 10 | 23 | 30 | 15 | Yes | No | Negative |
| 22 | 4 | 4 | 10 | 39 | 46 | 42 | Yes | No | D801N |
| 23 | 1 | 0 | 10 | 16 | 23 | 23 | No | No | E324Q |
| 24 | 4 | 1 | 10 | 30 | 31 | 30 | Yes | No | G755S |
| 25 | 7 | 3 | 10 | 33 | 33 | 30 | No | No | D801N |
| 26 | 3 | 9 | 10 | 35 | 35 | 26 | Yes | Yes | G947R |
| 27 | unknown | 19 | 19 | 25 | 25 | 6 | Yes | Yes | E815K |
| 28 | 1 | 0 | 10 | 25 | 26 | 26 | No | No | D801N |
| 29 | 1 | 0 | 10 | 20 | 20 | 20 | Yes | Yes | D801N |
| 30 | 1 | 1 | 10 | 21 | 21 | 20 | No | No | E815K |
| 31 | 3 | 0 | 2 | 12 | 14 | 14 | No | No | D801N |
| 32 | 1 | 0 | 2 | 5 | 5 | 5 | No | No | D801N |
| 33 | 1 | 1 | 3 | 4 | 4 | 3 | Yes | No | Val919del |
| 34 | 12 | 2 | 6 | 7 | 7 | 5 | Yes | No | c.993+1_993+2delTG |
| 35 | 4 | 0 | 2 | 9 | 9 | 9 | No | No | S137Y |
| 36 | 1 | 0 | 4 | 10 | 10 | 10 | No | No | D801N |
| 37 | 5 | 1 | 5 | 10 | 12 | 11 | No | No | D801N |
| 38 | 5 | 1 | 3 | 4 | 4 | 3 | Yes | No | G947R |
| 39 | 2 | 0 | 2 | 8 | 9 | 9 | No | No | D801N |
| 40 | 3 | 1 | 5 | 10 | 10 | 9 | No | No | D801N |
| 41 | 2 | 0 | 7 | 8 | 8 | 8 | Yes | No | E815K |
| 42 | 4 | 1 | 2 | 4 | 10 | 9 | No | No | D801N |
| 43 | 2 | 2 | 10 | 34 | 34 | 32 | No | No | Thr264_Ile289delinsIleLeuGly |
| 44 | 1 | 2 | 2 | 9 | 9 | 7 | Yes | Yes | L715P |
| 45 | 1 | 5 | 03 | 11 | 13 | 8 | Yes | Yes | A911P |
| 46 | 1 | 0 | 23 | 30 | 30 | 30 | Yes | No | D801N |
| 47 | 1 | 4 | 5 | 8 | 8 | 4 | Yes | No | D609Y |
| 48 | 1 | 0 | 2 | 6 | 6 | 6 | No | No | G974R |
| 49 | 6 | 6 | 8 | 9 | 11 | 5 | No | No | G89A |
| 50 | 3 | 1 | 2 | 3 | 4 | 3 | No | No | E828K |
| 51 | 1 | 1 | 6 | 17 | 18 | 17 | No | No | E815K |
| 52 | 1 | 0 | 6 | 17 | 17 | 17 | Yes | Yes | E815K |

**Supplementary Table 3.**

| **Behavioral Test** | **Critical Value (F,t or H depending on statistical test used)** | **Degrees of Freedom** | **Statistical Test Used** | **N** |
| --- | --- | --- | --- | --- |
| Balance Beam Traversal Time | t = -6.967 | 10 | Paired T test | 11 MT/WT Young; 11 MT/WT Adult |
| Balance Beam Hindlimb Slips | H = 4.263 | 1 | Kruskall Wallis | 11 MT/WT Young; 11 MT/WT Adult |
| ­ | t = -3.595 | 3 | Paired T test | 4 MT/WT Young; 4 MT/WT Adult |
| Rotarod | t = 0.0729 | 10 | Paired T test | 11 MT/WT Young; 11 MT/WT Adult |
| Open Field Crosses | t = 0.978 | 10 | Paired T test | 11 MT/WT Young; 11 MT/WT Adult |
| Time Spent in center of open field | t = -1.234 | 10 | Paired T test | 11 MT/WT Young; 11 MT/WT Adult |
| Forelimb Grip Strength | t = -1.485 | 10 | Paired T test | 11 MT/WT Young; 11 MT/WT Adult |
| Hindlimb Grip Strength | t = -2.096 | 10 | Paired T test | 11 MT/WT Young; 11 MT/WT Adult |
| Gait Forelimb Stride Length-Males | t = -1.409 | 4 | Paired T Test | 5 MT/WT Young; 5 MT/WT Adult |
| Gait Forelimb Stride Length-Females | t = -1.087 | 3 | Paired T Test | 4 MT/WT Young; 4 MT/WT Adult |
| Gait Hindlimb Stride Length-Males | t = -1.515 | 4 | Paired T Test | 5 MT/WT Young; 5 MT/WT Adult |
| Gait Hindlimb Stride Length-Females | t = -2.399 | 3 | Paired T Test | 4 MT/WT Young; 4 MT/WT Adult |
| Gait Forelimb BSO-Males | t = -0.676 | 4 | Paired T Test | 5 MT/WT Young; 5 MT/WT Adult |
| Gait Hindlimb BSO-Males | t = 2.421 | 4 | Paired T Test | 5 MT/WT Young; 5 MT/WT Adult |
| Gait Hindlimb BSO-Females | Z-Statistic (based on positive ranks)=1.461 | 3 | Wilcoxon Signed Rank Test | 4 MT/WT Young; 4 MT/WT Adult |
| Mortality | N/A | 1 | Fisher’s Exact | 16 Young; 5 Adult |
| Seizure Stage | N/A | 1 | Fisher’s Exact | 16 Young; 5 Adult |
| Hemiplegia | N/A | 1 | Fisher’s Exact | 16 Young; 5 Adult |
| Dystonia | N/A | 1 | Fisher’s Exact | 16 Young; 5 Adult |
| Seizure Duration | t=2.26644205 | 17 | T Test assuming unequal variances | 16 Young; 5 Adult |
| Novel Object | t=0.781 | 10 | Paired T Test | 11 MT/WT Young; 11 MT/WT Adult |

**Supplementary Figure 1**. EEG recordings from a four months old male *Mashl^+/-^* mouse before and after the forced swimming cold water (CW) induction test (Isaksen *et al.* 2017, Hunanyan *et al*. 2015, 2018). Mouse was placed in a 3-L beaker with 2 L water at 5 °C for 2 minutes and EEG was recorded from bilateral frontal motor cortex using bipolar electrodes one on each side (electrodes were inserted at the following coordinates: 0.2 mm anterior, 1.5 mm lateral, 1.5 mm below the dura). (A) Baseline recording before mouse exposure to CW showing baseline EEG background. (B) Recording at the end of the 2 minutes CW immersion directly after removal from water. On the exam the mouse’s left hindlimb was completely limp and hypotonic (duration ~ 40 seconds). EEG shows attenuation of the baseline EEG and no electrographic seizure activity. (C) Recording during dystonia (~3 minutes duration). Mouse was diffusely stiff in trunk and all four limbs in a distorted abnormal posture. EEG shows further attenuation of the background activity and still no electrographic seizure activity was observed. (D) EEG traces during seizure class V (duration ~ 50 minutes). EEG shows electrographic seizure activity of waxing and waning polyspike activity. (E) Post-ictal recording approximately 30 minutes following the end of the behavioral seizure, shows resumption of the background EEG after the mouse returned to baseline. Red traces show EEG recordings from the left cortex, black traces from the right cortex.

1. B. C.

D. E.
